# Increased respiratory drive relates to severity of dyspnea in systemic sclerosis

**DOI:** 10.1186/1471-2466-14-57

**Published:** 2014-04-04

**Authors:** Maarten K Ninaber, Willem BGJ Hamersma, Annemie JM Schuerwegh, Jan Stolk

**Affiliations:** 1 Department of Pulmonology (C3), Leiden University Medical Center, PO Box 9600, Leiden 2300RC, the Netherlands; 2 Rheumatology, Leiden University Medical Center, Leiden, the Netherlands

**Keywords:** Respiratory drive, Systemic sclerosis, Dyspnea evaluation

## Abstract

**Background:**

Dyspnea may be a presenting symptom in progressive systemic sclerosis (SSc). Respiratory drive (mouth occlusion pressure, MOP, at rest and during CO_2_ rebreathing, 7% CO_2_, 93% O_2_) is a major determinant of dyspnea and may relate to the magnitude of dyspnea.

**Methods:**

In a prospective design, MOP at 0.1 sec (P0.1) was measured in 73 SSc patients while breathing room air and during CO_2_ rebreathing. An abnormal V’E/P0.1 is defined as < 8 L/min/cm H_2_O. Dyspnea scores were assessed by a shortness of breath questionnaire (UCSD dyspnea scale).

**Results:**

Mean P0.1 in patients with normal V’E/P0.1 (n = 45) was 1.1 ± 0.04 and 1.6 ± 0.08 cm H_2_O in patients with abnormal V’E/P0.1 (n = 28), p <0.001. ∆P0.1/∆PetCO_2_ differed significantly between these groups (0.45 versus 0.75 cm H_2_O/mmHg, P < 0.001), but no significant difference was present in ∆V’E/∆PetCO_2_. V’E/P0.1 showed the highest significant correlation with the UCSD dyspnea score (r = -0.76, p <0.001). UCSD cut-off value for abnormal V’E/P0.1 was 8.5 (sensitivity 93%, specificity 96%, area under the curve 0.98).

**Conclusions:**

In SSc patients an abnormal V’E/P0.1 better relates to the severity of dyspnea than traditional lung function parameters and can easily be assessed at first outpatient consultation.

## Background

Systemic sclerosis (SSc) is a rare, heterogeneous condition of unknown etiology characterized by microvascular injury and deposition of excess collagen in skin and internal organs [1]. Principle subsets of SSc include limited cutaneous SSc (lcSSc) and diffuse cutaneous SSc (dcSSc) [2]. Importantly, progressive systemic sclerosis may involve interstitial lung disease (ILD) resulting into a restrictive lung function pattern abnormality [1]. In addition, pulmonary arterial hypertension (PAH) may arise in the course of the disease [1]. Validated measures to monitor progression of SSc are necessary for clinical trials and routine care of patients with SSc. Dyspnea as a presenting symptom occurs in 20% of all newly diagnosed SSc patients and 70% of patients with SSc complicated by ILD or PAH patients complain of dyspnea [1]. Importantly, early recognition of disease progression related to organ damage and initation of treatment may improve health-related outcomes. Lung volumes and gas transfer studies are related to disease severity in SSc [1] and used for initation of treatment and evaluation. However, whether the magnitude of dyspnea relates to these pulmonary function tests is not known.

The impedance of the respiratory system is influenced by lung and chest wall compliance and respiratory flow resistance [3]. An increased respiratory impedance is recognized as the most frequent cause of dyspnea [3]. Other respiratory abnormalities resulting into dyspnea may include hypoxia, respiratory muscle weakness or pulmonary vasculopathy (pulmonary embolisms, pulmonary arterial hypertension). In progressive SSc, dyspnea may arise from an increased impedance of the respiratory system caused by ILD. In SSc, ILD or limited chest wall excursions due to a thickened thoracic skin is considered to cause this increased impedance [4,5]. Furthermore, dyspnea may result from an inappropriate ventilatory response upon the chemoreflex drive at rest and to hypercapnia [6,7].

To assess the ventilatory output as an index of the respiratory drive, resting ventilation (V’E) and tidal volumes can be evaluated [8]. Ventilation, however, is an imperfect output parameter of the respiratory drive since it is affected by alterations in the impedance of the respiratory system (i.e. mechanical properties of the lung and chest wall) independently of changes in respiratory sensitivity to hypercapnia [3,6,9]. To assess the respiratory drive, mouth occlusion pressures (MOP) as an index of the output can be measured [3]. Important advantages of this technique include the reproducibility within each subject and reported values independent of age [10]. In a study of normal subjects and patients with ILD, a V’E/P0.1 greater than 8 l/min/cm H_2_O sharply separated a normal from an abnormal response [10]. Therefore, in patients who report dyspnea and who have concomitant ILD, a low V’E to a high P0.1 (i.e. low V’E/P0.1) is expected.

In addition to V’E/P0.1 at rest, the respiratory drive to hypercapnia (P0.1 to CO_2_, i.e. ∆P0.1/∆PetCO_2_) provides insight into the central chemoreflex drive to hypercapnia [11-13]. In patients with ILD and dyspnea, the central chemoreflex drive may result into a falsely low ventilatory response to hypercapnic stimulation [7]. To overcome this, the central chemoreflex drive to hypercapnia may be assessed by mouth occlusion pressures (∆P0.1/∆PetCO_2_) [6,11,12].

Based on the above we hypothesized that in patients with SSc the respiratory drive, as measured by P0.1, V’E/P0.1 and mouth occlusion pressures to CO_2_ rebreathing, may better relate to the magnitude of reported dyspnea than the severity of gas transfer or lung volume impairment as measured by PFTs. Furthermore, we hypothesized that the respiratory drive to hypercapnia is increased in SSc patients who reported dyspnea.

## Methods

### Patients

We prospectively screened SSc patients referred to an outpatient targeted health care program. All patients underwent an intensive screening procedure which included PFTs, serum laboratory testing, echocardiography, high-resolution chest CT scanning (HRCT) and a cardiopulmonary exercise test (CPET). Furthermore, all patients consulted a rheumatologist, cardiologist and a pulmonologist. All tests were done in one or two consecutive days. Patients were classified as limited systemic sclerosis (lcSSc) or diffuse systemic sclerosis (dcSSc) according to the LeRoy criteria [2].

## Ethics

The local Medical Ethical Committee of the Leiden University Medical Center approved the protocol. A written informed consent was obtained from each patient prior to enrollment.

### Standard pulmonary function testing

PFTs were measured in all SSc patients including spirometry and gas transfer studies and expressed as percentage predicted [14,15]. Total lung capacity (TLC) was measured by the multiple breath helium dilution method [14] and diffusion capacity for carbon monoxide (DLCO) by the single breath carbon monoxide method [15].

### Measuring mouth occlusion pressures during resting ventilation and CO_2_ rebreathing

Subjects were seated comfortably, attached to the mouthpiece with a noseclip in place. At randomized intervals, and without the subject’s knowledge, the inspiratory side of the rebreathing circuit was occluded during late expiration. The pressure generated at 0.1 s after the onset of inspiration was obtained in each subject during several minutes with a minimum of 10 measurements prior to the rebreathing test [3]. Thereafter, occlusion pressures were measured simultaneously during CO_2_ rebreathing at randomized intervals [3,16]. The slope of this curve was used as an index of the respiratory drive to hypercapnia (i.e. central chemoreflex drive) and reported as ΔP0.1/ΔPetCO_2_[3,16].

### Measuring the hyperoxic ventilatory response to hypercapnia (HCVR)

We used a simple rebreathing technique according to Read’s rebreathing technique, which consisted of a rebreathing bag filled with a gas mixture (7% CO_2_ and 93% O_2_) [17]. In the rebreathing bag, a total volume of approximately twice the measured vital capacity of the patient was used.

Under hyperoxia the ventilatory response to hypercapnia (HCVR) represents the central chemoreflex response only, assuming that the peripheral chemoreflex drive is suppressed by hyperoxia [16,17]. Equilibrium of pressures between CO_2_ in cerebral blood and end-tidal PCO_2_ exhalation at the mouth (PetCO_2_) is expected not to occur before recirculation of cerebral blood flow [17]. Respiratory volumes were recorded by a turbine volume measuring device (Oxycon-Pro, Jaeger). The Oxycon Pro was calibrated according to the instruction manual before each test (Oxycon instruction manual ver. 4.5. Erich Jaeger GmbH, Hoechberg, Germany) [18]. Oxygen and CO_2_ analyzers were calibrated with room air and certified calibration gases at 180 kPa (16% O_2_, 5% CO_2_ and 79%N_2_). The flow turbine (Triple V, Erich Jaeger GmbH, Hoechberg, Germany) was calibrated with a 3.00 liter 5530 series calibration syringe (Hans Rudolph, Inc, Kansas City, USA). Both gas and volume calibration were repeated until the difference between consecutive calibrations was less than 1%. Therefore, measurements were not considered to be influenced under hyperoxia. Expired gas at the mouth was sampled continuously and analyzed for PetCO_2_ by a fast-response infrared analyzer. The software calculated tidal volumes, inspiratory and expiratory times, minute ventilation, and PetCO_2_ on a breath-by-breath basis. The hyperoxic ventilatory response to hypercapnia (HCVR) was measured during several minutes after equilibrium between the end-tidal CO_2_ and mixed venous CO_2_. In this phase, a linear increase in V’E with respect to PetCO_2_ was observed. The slope of this curve was used as the index of the ventilatory central chemosensitivity and reported as ΔV’E/ΔPetCO_2_[17].

### UCSD shortness of breath questionnaire

We used a previously validated shortness of breath questionnaire which evaluates in 24-items self-reported shortness of breath while performing a variety of activities of daily living [19]. It was administered by MKN prior to the MOP and rebreathing study while the subject sitting comfortably.

### Statistical analysis

Statistical analysis was performed with the SPSS 20.0 package (SPSS, Inc., Chicago, IL, USA). Continuous variables are expressed as mean value ± standard deviations. P values < 0.05 were considered significant. Categorical data are presented as frequencies and percentages. Statistical comparisons were performed by using Student’s T-test for continuous variables, and chi square test for binary variables. Correlations between clinical parameters, pulmonary function tests, were expressed in terms of Pearson’s or Spearman’s correlation coefficient when appropriate. The receiver operating characteristic (ROC) curve was used to evaluate the optimal cut-off value for the UCSD dyspnea score in relation to an abnormal V’E/P0.1.

## Results

In total, 73 SSc patients were prospectively evaluated by measuring PFTs, MOP and the hyperoxic ventilatory response to hypercapnia (HCVR). Patients were classified by their V’E/P0.1 according to Scott GC and Burki NK [10], where ≥ 8 l/min/cmH_2_O was defined as normal.

Anthropometric and lung function data are presented in Table 1. In the group with a V’E/P0.1 ≥ 8 L/min/cmH20, lcSSc patients were significantly more present. In all patients with an abnormal V’E/P0.1 (< 8 L/min/cmH_2_O), spirometric and gas transfer studies were significantly lower. All SSc patients were normocapnic at rest (PaCO_2_ = 5.24 ± 0.41 kPa).

**Table 1:** **Anthropometric and pulmonary function data of 73 SSc patients classified by V’E/P0.1 ≥ 8 l/min/cmH**_**2**_**O**[10]

	**V’E/P0.1 < 8 l/min/cmH**_**2**_**O N = 28**	**V’E/P0.1 ≥ 8 l/min/cmH**_**2**_**O N = 45**	P value*
Age (yrs)	54.4 (11.8)	50.9 (14.4)	0.25
Female sex (%)	26 (93)	37 (82)	0.11
Height (cm)	164 (7.2)	171 (8.7)	0.002
Weight (kg)	67.1 (15.3)	69.8 (12.8)	0.43
BMI (cm/kg2)	24.8 (5.4)	23.9 (3.7)	0.48
lcSSc subtype (%)	10 (36)	34 (76)	0.02
PaO_2_ (kPa)	10.4 (0.36)	10.6 (0.29)	0.58
PaCO_2_ (kPa)	5.31 (0.41)	5.18 (0.43)	0.25
FVC (% pred)	92.1 (19.9)	111.1 (19.7)	0.001
DLCOc SB (% pred)	57.7 (15.8)	70.7 (15.1)	0.001
TLC-He (% pred)	81.5 (16.6)	95.4 (13.5)	<0.001
FRC/TLC (%)	53 (17)	56 (7)	0.23

*P value expressed for student T-test.

*PaO*_*2*_*(partial arterial oxygen pressure, kPa).*

*PaCO*_*2*_*(partial arterial carbon dioxide pressure, kPa).*

FVC (forced vital capacity, % pred).

DLCOc SB (diffusion capacity for carbon monoxide single breath, % pred).

TLC-He (total lung capacity, helium-dilution, % pred).

FRC/TLC (ratio of functional residual capacity and TLC, %).

Indices of MOP differed significantly between the two groups (Table 2). In contrast, there was no difference in the hyperoxic ventilatory response to hypercapnia. In our study, patients with a normal V’E/P0.1 had a mean ∆P0.1/∆PetCO_2_ of 0.45 ± 0.19 cmH_2_O/mmHg, which differed significantly from patients with an abnormal V’E/P0.1. The latter group had an increased respiratory drive to hypercapnia (mean ∆P0.1/∆PetCO_2_ 0.75 ± 0.42 cmH_2_O/mmHg).

**Table 2:** **Indices of MOP, CO**_**2 **_**rebreathing and the UCSD dyspnea score**[18]**of 73 SSc patients**

	**V’E/P0.1 < 8 l/min/cmH**_**2**_**O N = 28**	**V’E/P0.1 ≥ 8 l/min/cmH**_**2**_**O N = 45**	P value*
UCSD SOBQ	13 (3.5)	2.2 (2.7)	<0.001
P0.1	1.60 (0.57)	1.10 (0.31)	<0.001
V’E	8.7 (2.3)	11.6 (3.5)	<0.001
V’E/P0.1	5.68 (1.24)	10.83 (2.46)	<0.001
∆V’E/∆PetCO_2_	2.31 (2.0)	2.49 (1.76)	0.71
(∆V’E/FVC)/∆PetCO_2_	0.93 (0.72)	0.57 (0.51)	0.07
∆P0.1/∆PetCO_2_	0.75 (0.42)	0.45 (0.19)	0.01

*P value expressed for student T-test.

UCSD SOBQ (shortness of breath questionnaire; score range 0–120).

*P0.1 (mouth occlusion pressure at 0.1 second; cmH*_*2*_*O).*

V’E (minute ventilation; L/min).

*V’E/P0.1 (central inspiratory neuromuscular respiratory drive; L/min/cmH*_*2*_*O).*

*∆V’E/∆PetCO*_*2*_*(hyperoxic ventilatory response to hypercapnia; L/min/mmHg).*

*(∆V’E/FVC)/∆PetCO*_*2*_*(hyperoxic ventilatory response to hypercapnia, corrected for FVC; FVC/min/mmHg).*

*∆P0.1/∆PetCO*_*2*_*(slope of occlusion pressure to hypercapnia; cmH*_*2*_*O/mmHg).*

**Figure 1:**
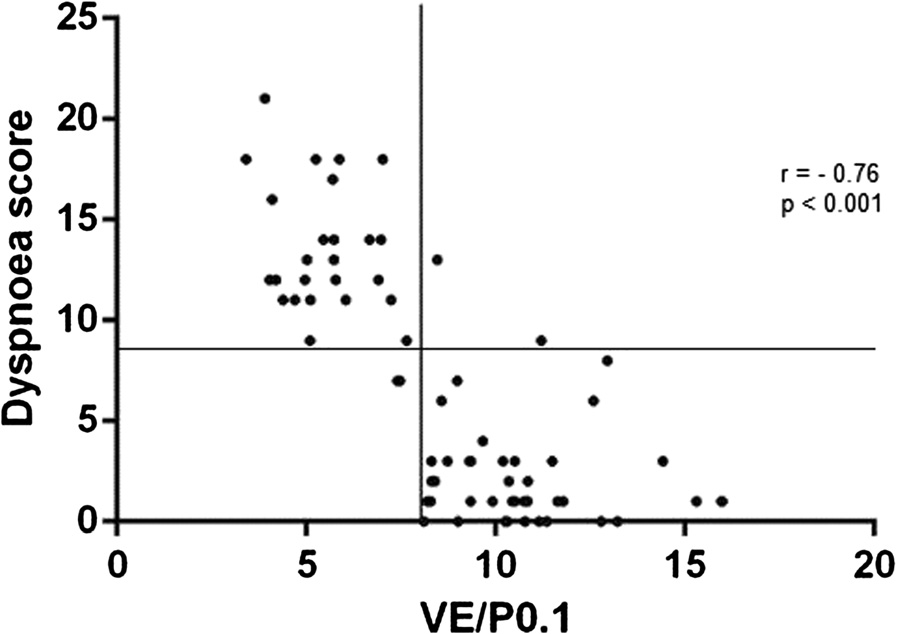
**Correlation between the UCSD dyspnea score [**[18]**] and V’E/P0.1 in 73 prospectively screened SSc patients.** Vertical line set at V’E/P0.1 = 8 L/min/cmH_2_O. Horizontal line set at UCSD 8.5.

All pulmonary function test parameters correlated inversely and significantly with the UCSD shortness of breath questionnaire (coefficient of correlation ranging from -0.39 to -0.49). V’E/P0.1, however, showed the highest significant correlation with the UCSD dyspnea score (r = -0.76, p < 0.001, Figure 1). The cut-off value in UCSD dyspnea score for an abnormal V’E/P0.1 was 8.5 (sensitivity 93%, specificity 96%, area under the curve 0.98).

## Discussion

We report that in SSc patients with an abnormal inspiratory respiratory drive (V’E/P0.1 < 8 L/min/cmH_2_O) the sensation of dyspnea as measured by the UCSD dyspnea score differed from SSc patients with a normal respiratory drive. In addition, with a cut-off value of 8.5, the dyspnea score showed a high sensitivity and specificity for an abnormal V’E/P0.1. Furthermore, an abnormal V’E/P0.1 better relates to the magnitude of dyspnea than traditional lung function parameters and in these SSc patients an increased central chemoreflex drive to CO_2_ is present. Therefore, at first outpatient consultation, SSc patients complaining of dyspnea can easily be classified by using mouth occlusion pressures.

We showed that the level of dyspnea perception measured by the UCSD shortness of breath questionnaire [19] had a strong inverse correlation with the inspiratory neuromuscular drive as measured by V’E/P0.1 (Figure 1). A high dyspnea score and an increased respiratory drive to hypercapnia suggest that the work of breathing is increased due to an increased respiratory impedance [7,10]. An increased activation of central respiratory centers results not only into an increase in minute ventilation but also into an increased perception of dyspnea [20]. The basis for this awareness originates from the exchange in information between the motor and sensory cortex and is referred to as corollary discharge [20]. Although the work of breathing is not the sole cause of dyspnea, increased effort as a result from an increased mechanical load causes a heightened sense of respiratory effort. Consequently, this may explain the strong correlation between the dyspnea score and the respiratory drive in the present study. This drive can be easily measured using mouth occlusion pressures at rest. As our results indicate, it better relates to dyspnea than impairment in lung volumes or gas transfer and may be present before significant impairment in these function tests arises. Therefore, measurement of the neuromuscular inspiratory drive may function in clinical practice in SSc patients complaining of dyspnea as a screening tool for detection of ILD or PAH.

Ventilation and its components, tidal volume and breathing frequency, depend on the compliance of the respiratory system and airway resistance [3,9-11]. Importantly, one would like to differentiate patients who will not breathe because of central or neuromuscular inadequacy from those who cannot breathe because of mechanical abnormalities of the chest. The ventilatory response to hypercapnia is known for its large variability and reported responses may vary from 2.0 to 4.7 L/min/mmHg [3,17]. Although a correction by FVC will lead to less variability (reported values vary from 0.42 to 0.94 FVC/min/mmHg [3]), measuring the respiratory drive in patients affected by ILD using ventilatory parameters remains troublesome.

The mouth occlusion pressure generated by the inspiratory muscles at functional residual capacity (P0.1) has been proposed as an useful test to avoid these disadvantages [3,9-11]. It is independent of flow resistance and respiratory compliance and less variability is observed in various subjects [3,10]. Taken this together, we argue that ventilation and its components, may not represent an accurate output of the respiratory drive in our patients.

In daily practice, measuring P0.1 is very simple to apply and joined with a rebreathing bag, measurement of CO_2_ responsiveness is possible. In normal subjects, P0.1 values of 0.75-1.5 cmH_2_0 have been described [3,10]. In addition, normal V’E/P0.1 is defined by ≥ 8 L/min/cmH_2_O, as evaluated by Scott and Burki [10]. The responsiveness to hypercapnia by measuring P0.1 (∆P0.1/∆PetCO_2_) in normal subjects is reported to be 0.17-0.49 cmH_2_O/mmHg [3,16]. This is in agreement with our results of SSc patients with a normal V’E/P0.1 (Table 2, mean ∆P0.1/∆PetCO_2_ 0.45 cmH_2_O/mmHg). Thus, in these SSc patients, the respiratory compliance and airflow resistance did not affect occlusion pressures or the responsiveness to hypercapnia and is therefore considered to be normal. Furthermore, our data of mouth occlusion pressures during resting minute ventilation and during CO_2_ rebreathing in patients with normal V’E/P0.1 are consistent with those of others [3,7,10,21]. However, in the present study no significant difference was seen in ∆V’E/∆PetCO_2_ between the groups as classified by V’E/P0.1 (Table 2). Since there was a significant difference present between these groups using mouth occlusion pressures, minute ventilation may not represent the neuromuscular drive adequately in SSc patients with an increased respiratory impedance.

Although our reported values of the respiratory drive, as measured by P0.1, were approximately similar, differences from those previously reported by Walterspacher et al. are present [21]. Potential reasons may include the differences in degree of restrictive lung function as measured by FVC% predicted. Our SSc patients with an abnormal V’E/P0.1 had mildly reduced FVC% predicted and may have preserved inspiratory muscle function. Furthermore, our SSc patients did not avoid activities provoking dyspnea resulting into deconditioning and muscle wasting. Secondly, they did not use immunosuppressive agents affecting respiratory muscle function (prednisone). Finally, polyneuropathy, which may cause an impaired respiratory muscle function, was not likely in our patients. Therefore, respiratory drive was considered to be appropriate in our SSc population.

In our study, patients with a low V’E/P0.1 had an increased responsiveness to hypercapnia which suggests an increased central respiratory drive (Table 2). This group was characterized by fewer limited SSc patients, a lower forced vital capacity, more impaired gas transfer and a lower lung volume (Table 1). Importantly, the majority of these patients had evidence of interstitial lung disease on their HRCT and some patients had an elevated tricuspid insufficiency gradient (data not shown). The disease duration did not differ between these groups. The difference in V’E/P0.1 between the groups are related to SSc disease severity and therefore influence the impedance of the respiratory system. Consequently, differences in V’E/P0.1 were observed.

Similar results are reported by Gorini and coworkers in normocapnic ILD patients without SSc [7]. This contrasts to the concept of gradual down-regulation in central respiratory sensitivity for carbon dioxide in scleroderma patients [4]. DiMarco and coworkers concluded that in patients with ILD, non-chemical, and presumably neural, mechanisms, both increase respiratory drive and alter the breathing pattern [22].

Several factors may influence the central sensitivity to CO_2_ as measured by the ventilatory response to hypercapnia, such as the hormonal status, sex, age, use of sedative agents and caffeine [8,17]. However, these factors will generally not result into an abnormal V’E/P0.1 since the compliance of the respiratory system or airway resistance is not affected.

Some considerations may apply to our study. First, we evaluated MOP and CO_2_ rebreathing in patients with systemic sclerosis. We used a dataset of normal values for normal resting minute ventilation as a function of occlusion pressures (V’E/P0.1) [10]. As reported by others a value higher than 8 L/min/cmH_2_O, independent of age or sex, identifies subjects with normal PFTs and therefore provides a reliable index of respiratory drive [3,10]. Consequently, we restricted our measurements in a patient group, only classified by V’E/P0.1. Our study contained 73 patients, which was similar to previous studies evaluating mouth occlusion pressures [3,10], and considered to be sufficient for group difference statistics. Despite the relatively mild range in low dyspnea scores (range 0–21; Figure 1), a significant difference in V’E/P0.1 and ∆P0.1/∆PetCO_2_ was present (Table 2), indicating the sensitivity of the mouth occlusion pressure test. Furthermore, assessing V’E/P0.1 to an additional load such as exercise may have increased this difference since airflow resistance will increase and consequently the impedance of the respiratory system.

Secondly, we did not measure the sensation of dyspnea, as assessed at rest by the UCSD questionnaire, during CO_2_ rebreathing. To evaluate the relationship between the intensity of dyspnea and respiratory chemosensitivity may have strengthen our results, however this questionnaire was not designed to be administered during a rebreathing or exercise test.

## Conclusions

In summary, the results of the present study show that patients with systemic sclerosis display a substantial variability in the ventilatory response to hypercapnia. In contrast, mouth occlusion pressures, independent of respiratory compliance or airflow resistance, provide an accurate outcome parameter in combination with minute ventilation as an initial evaluation for mild dyspnea. They better relate to the magnitude of patient reported dyspnea than traditional lung function parameters such as FVC or DLCO% predicted. Furthermore, in our SSc patients with an abnormal V’E/P0.1, an increased respiratory drive to hypercapnia was present. Since dyspnea is one of the most frequently reported symptoms at first consultation in SSc, an easy test to discriminate between normal or abnormal respiratory mechanics would be obligatory. Therefore, we suggest that an abnormal V’E/P0.1 in combination with reported dyspnea indicates further assessment of respiratory involvement in SSc patients.

## Competing interests

The authors declare they have no competing interests.

## Authors’ contributions

MKN, WBGJH, AJMS and JS designed the study. MKN, WBGJH, AJMS, JS all contributed to the clinical work for the study. MKN, WBGJH and JS analyzed the data and wrote the manuscript. All authors read and approved the final manuscript.

## Pre-publication history

The pre-publication history for this paper can be accessed here:

http://www.biomedcentral.com/1471-2466/14/57/prepub
